# Case report: Pheochromocytoma-induced pseudo-Cushing’s syndrome

**DOI:** 10.3389/fendo.2024.1491873

**Published:** 2024-12-16

**Authors:** Małgorzata Bobrowicz, Anna Nagórska, Anna Karpiłowska, Marek Rosłon, Joanna Hubska, Adrianna Gładka, Sadegh Toutounchi, Łukasz Koperski, Urszula Ambroziak

**Affiliations:** ^1^ Department of Internal Medicine and Endocrinology, University Clinical Centre of the Medical University of Warsaw, Warsaw, Poland; ^2^ Doctoral School of Medical University of Warsaw, Warsaw, Poland; ^3^ Department of General, Endocrine, and Vascular Surgery, Medical University of Warsaw, Warsaw, Poland; ^4^ Department of Pathology, Medical University of Warsaw, Warsaw, Poland

**Keywords:** pseudo-Cushing’s syndrome, ectopic ACTH secretion, hypercortisolaemia, pheochromocytoma, cachexia

## Abstract

Non-neoplastic hypercortisolaemia, also known as pseudo-Cushing’s syndrome (PCS), is a physiological overactivation of the hypothalamic–pituitary–adrenal axis that can be triggered by conditions such as depression, eating disorders, extreme exercise, obesity, alcoholism, poorly controlled diabetes, chronic kidney disease, and cachexia. Here, we describe an unusual case of pheochromocytoma-induced PCS. A 66-year-old woman was referred to the hospital due to pronounced weakness, loss of appetite, apathy, weight loss, newly diagnosed diabetes mellitus, and poorly controlled hypertension. The biochemical evaluation suggested ACTH-dependent hypercortisolemia with severe hypokalemia, metabolic alkalosis, and hyperglycemia. Markedly elevated levels of metanephrines, along with imaging showing a heterogeneous adrenal lesion, provided evidence for pheochromocytoma. Considering the clinical features and the results of laboratory and imaging tests, there was a suspicion of hypercortisolemia due to ectopic ACTH secretion by a pheochromocytoma. The patient underwent adrenalectomy following pre-treatment with doxazosin and metyrapone, enteral feeding, protein supplementation, and insulin administration. Post-surgery, the patient did not require further antidiabetic medication, experienced gradual weight gain, improved well-being, and did not need glucocorticoid supplementation. Histopathological examination confirmed a pheochromocytoma; however, both anti-ACTH and anti-CRH stainings were negative, leading to a diagnosis of PCS. This case highlights the distinctive presentation of PCS caused by pheochromocytoma, as demonstrated through clinical, laboratory, and histopathological findings, and emphasizes the successful resolution achieved through adrenalectomy and supportive care.

## Introduction

Pseudo-Cushing’s syndrome (PCS), also referred to as non-neoplastic hypercortisolism, results from the physiological overactivity of the hypothalamic-pituitary-adrenal (HPA) axis (1). PCS can be triggered by conditions such as depression, eating disorders, severe physical stress, obesity, insulin resistance, chronic kidney disease, and chronic alcoholism (2). Although the clinical manifestations of PCS may vary between patients, it is generally accepted that their onset is rather slow (3). Importantly, the differentiation between PCS and CS is a diagnostic challenge even for experts in the field (4).

The mechanisms contributing to overactivation of the HPA axis have been elegantly reviewed by Scaroni et al. (5). Briefly, lower levels of cortisol-deactivating enzymes 5-α-reductase and 11β-hydroxysteroid dehydrogenase type 2 have been reported in patients with neurophychiatric disorders, reduced cortisol clearance, accompanied by changes in the affinity of cortisol to corticosteroid-binding globulin, elevation of corticotropic hormone (CRH) levels with resistance to glucocorticoids in patients with eating disorders, and increased secretion of CRH and increased activity of 11-βHSD type 1 in obese and alcohol-dependent individuals. PCS related to severe malnutrition has been rarely reported (6, 7).

Distinguishing PCS from Cushing syndrome (CS), including ectopic ACTH secretion (EAS), can be difficult due to their similar clinical and biochemical presentations (8). Hence, appropriate diagnosis is crucial to implement relevant treatment. Accurate decisions reduce the mortality risk associated with hypercortisolemia and preserve patient from the risk and complications associated with unnecessary procedures.

EAS is a paraneoplastic syndrome responsible for 10-20% of all cases of CS. CRH secretion by the tumor as well as CRH and ACTH co-secretion have been reported incidentally (2). It is estimated that 70% of EAS cases arise from chest tumors, with small-cell carcinomas and bronchial carcinoids being the most frequent. The other 10% to 15% are attributed to neuroendocrine tumors of the pancreas, while other rare sources include medullary thyroid cancer, pheochromocytoma, and others (9). The molecular mechanisms leading to EAS, as well as other small molecule compounds in paraneoplastic symptoms, are not fully understood (10). To date, epigenetic mechanisms, especially POMC promoter hypomethylation, have been reported in some EAS-inducing tumors (11, 12). Additionally, tumor-specific expression of transcription factors favoring ACTH production has been suggested (13, 14).

The clinical picture of EAS is heterogeneous and depends of the original cause (15). In general, two phenotypes of EAS can be observed: one associated with overt, mostly incurable malignancies, exemplified by small-cell lung cancer (SCLC), and the other associated with occult neoplasm, represented by bronchial carcinoma. While the first group is characterized with an atypical presentation dominated by muscle wasting, malignancy-induced weight loss and electrolyte and metabolic abnormalities, hyperpigmentation and rapid onset of symptoms (3-6 months), the second group exhibits characteristics of overt CS, a more slow (>6 months) development of symptomatic disease that needs to be differentiated with Cushing disease (16).

Pheochromocytomas account for approximately 5% of EAS cases (17). Extremely rare CRH-secreting pheochromocytomas leading to ectopic CS have also been reported (18). A meta-analysis by Elliot et al. (18) revealed that the vast majority (89%) of patients with ACTH-secreting or CRH-secreting pheochromocytomas presented with the characteristic cushingoid phenotype with moderate or severe hypercortisolism. Hypokalemia in these patients was more common than in patients with pituitary-dependent CS, and the degree of hypertension was more severe than in the general population of patients with pheochromocytoma as well as in patients with EAS due to other types of tumors. The frequency of diabetes (54%) almost doubled the rate reported in the general population of pheochromocytoma patients (18). Interestingly, Terzolo et al. (19) reported a case of cyclic CS in a woman with ACTH-secreting pheochromocytoma, suggesting a complexity of possible manifestations in EAS.

While ACTH-dependent hypercortisolemia in patients with pheochromocytoma is very likely caused by EAS or a coexistence of corticotroph adenoma, the possibility of PCS also has to be taken into consideration in the differential diagnosis. Herein, we present a unique case of pheochromocytoma-induced PCS evidenced by clinical, laboratory, and histopathological findings, and underscore the successful resolution through adrenalectomy and supportive care.

## Case report

**Figure 1:**
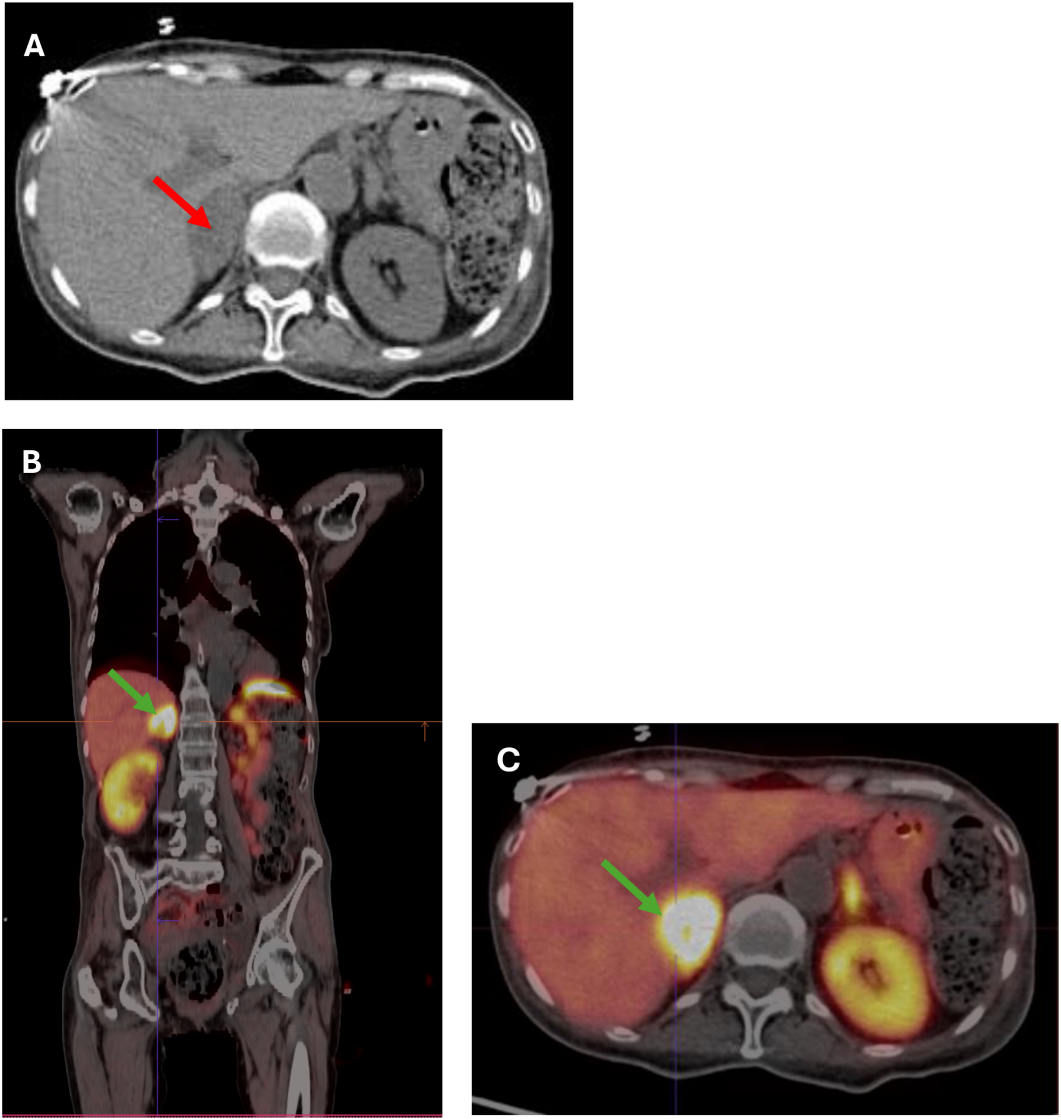
**(A)** CT scan showing a 32x35 mm heterogeneous, well-demarcated litho-cystic lesion (red arrow) in the right adrenal gland. **(B, C) (B)** Whole-body [68Ga] DOTATATE PET-CT image showing a hypodense right adrenal nodule measuring 36x22 mm (SUV max 35.4) (green arrow). **(C)** Cross-section [68Ga]Ga-DOTA-TATE PET/CT image showing a hypodense right adrenal nodule 36x22 mm (SUV max 35.4) (green arrow).

A 66-year-old woman was admitted to the county hospital due to pronounced weakness, loss of appetite, apathy, and a weight loss of 5 kg within one month, along with newly diagnosed diabetes mellitus and hypertension. The patient had a history of spells; she reported high blood pressure accompanied by heart palpitations and headaches. Due to rapid weight loss, and a suspicion of malignancy, chest and abdominal computed tomography (CT) was performed. The CT revealed a heterogeneous, well-demarcated litho-cystic lesion (32x25 mm) in the right adrenal gland with inhomogeneous contrast enhancement (**Figure 1A**). Hormonal results suggested ACTH-dependent hypercortisolemia with ACTH of 535 pg/ml, morning cortisol of 94 µg/dl, and evening cortisol of 85 µg/dl. Persistent hypokalemia (serum potassium of 2 mmol/l) was observed. Due to these multiple abnormalities, the patient was referred to the endocrine department for further diagnosis and treatment.

On admission to the clinic, the patient was in a severe general condition, recumbent, and cachectic (body weight of 35 kg; body mass index 15.6 kg/m²). She presented with impaired consciousness and a labile mood. Blood pressure was borderline (138/80 mmHg), and tachycardia (heart rate of 120 beats per minute) was observed. Apart from slight redness of the skin of the face and neckline (plethora), no other phenotypic features of hypercortisolemia were noted. Laboratory tests confirmed ACTH-dependent hypercortisolemia with a high plasma ACTH level, high plasma cortisol levels with loss of diurnal rhythm, elevated 24-hour urinary free cortisol excretion, high plasma dehydroepiandrosterone sulfate (DHEA-S) and testosterone levels, and significantly elevated levels of serum metanephrines (**Table 1**).

**Table 1:** Hormonal workup at diagnosis.

Parameter	Value	Reference range
ACTH	476.00	7.20-63.30 pg/ml
Morning plasma cortisol	125.00	4.82-19.50 ug/dl
Midnight plasma cortisol	137.00	<1.8 ug/dl
Urinary free cortisol	>239	4.30-176.00 ug/dl
DHEAS	337.00	9.40-246.00 ug/dl
Testosterone	6.19	0.10-1.42 ug/dl
Metanephrine	1194.23	<88.00 pg/ml
Normetanephrine	965.94	<200.00 pg/ml
3-metoxythyramine	52.27	<17.00 pg/ml

In the initial differential diagnosis, the coexistence of pheochromocytoma with ACTH-dependent hypercortisolemia or ectopic ACTH production by pheochromocytoma was considered. Due to the possibility of a pituitary tumor, an MRI was performed, showing no lesions in the pituitary gland. Subsequently, a [68Ga] DOTATE PET-CT scan was performed, which showed a hypodense right adrenal nodule measuring 36 x 22 mm, probably litho-fluidic with heterogeneous but high somatostatin receptor expression (SUV max 35.4) (**Figures 1B, C**).

**Figure 2:**
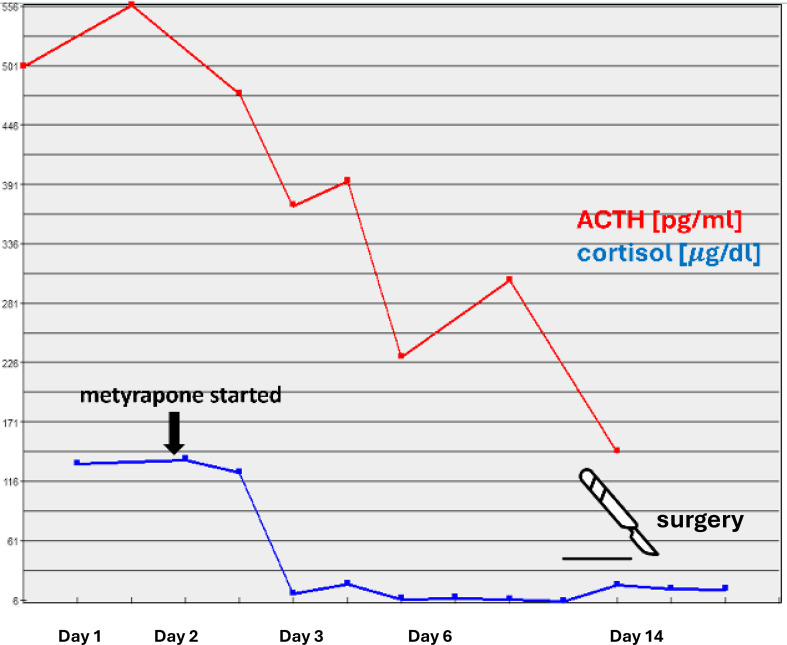
Serum levels of ACTH and cortisol following metyrapone administration.

Due to a strong suspicion of ACTH-producing pheochromocytoma, the patient was qualified for right adrenalectomy and prepared with increasing doses of doxazosin and a low dose of bisoprolol, potassium supplementation, parenteral hydration, protein supplementation, and intense insulin titration. The steroidogenesis inhibitor metyrapone (3x250 mg p.o. daily) was started, resulting in a rapid decrease in cortisol levels, leading to transient adrenal insufficiency requiring administration of 15 mg hydrocortisone daily (**Figure 2**). A rapid decrease in ACTH was observed already after first 24h of metyrapone (**Figure 2**) Intriguingly, a marked decrease in the levels of metanephrines (metanephrine to 749.35 pg/ml and normetanephrine to 593.55 pg/ml) was also observed after 12 days of metyrapone treatment.

After surgery, no clinical features nor laboratory results suggestive of adrenal insufficiency were observed (**Table 2**). In the 2-month period following adrenalectomy, the patient noted a gradual, slow increase in body weight (up to a BMI of 18.7 kg/m²) and improvement in well-being. Diabetes resolved, and hypertension control was optimal with a single dose of bisoprolol. Normalization of the levels of metanephrines (**Table 2**) confirmed the complete resection of the pheochromocytoma. From the patient’s perspective the patient feeled relieved after the resection of the tumor, she undergoes regular follow-up in the clinic where a constant improvement of her well-being and quality of life is observed.

**Table 2:** Hormonal workup performed two months after adrenalectomy.

Parameter	Value	Reference range
ACTH	55	7.20-63.30 pg/ml
Morning plasma cortisol	17.30	4.82-19.50 ug/dl
Midnight plasma cortisol	5.98	<1.8 ug/dl
DHEAS	15.20	9.40-246.00 ug/dl
Testosterone	0.11	0.10-1.42 ug/dl
Metanephrine	18.99	<88.00 pg/ml
Normetanephrine	114.98	<200.00 pg/ml
3-metoxythyramine	<LOQ	<17.00 pg/ml

**Figure 3:**
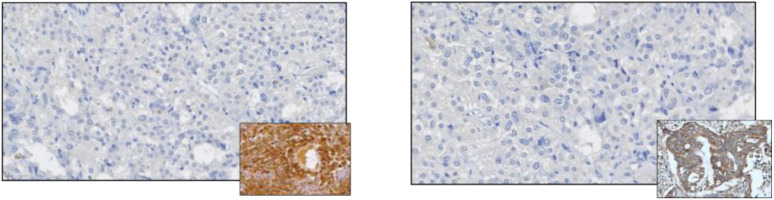
Negative immunostaining for ACTH (left panel) and negative immunostaining for CRH. Positive controls are shown in the miniatures.

The histopathological examination confirmed a diagnosis of pheochromocytoma, pT1 NX (according to AJCC, 8th ed.), PASS system - 3 points, GAPP system - 2 points (highly differentiated type). Immunohistochemistry (IHC) tests showed both negative anti-ACTH and anti-CRH stainings, thus the diagnosis of ectopic ACTH-dependent hypercortisolemia was finally rebutted (**Figure 3**). SDHB staining was focally positive with a weak cytoplasmic reaction (final status determination will be possible based on molecular/genetic tests). The rest of the excised adrenal did not show abnormalities, including the features of hyperplasia The patient was reluctant to perform further genetic investigations.

Rapid resolution of symptoms and normalization of laboratory parameters, no apparent cushingoid features at the diagnosis, and no signs of adrenal insufficiency post-surgery, together with negative stainings for ACTH and CRH, allowed the final diagnosis of pheochromocytoma-induced PCS. Factors contributing to such intense manifestations of pheochromocytoma are yet to be identified.

## Discussion

To the best of our knowledge, no reports on PCS induced by pheochromocytoma have been published before. The presented case underscores the unique manifestation of these two coexisting conditions, evident through clinical, laboratory, and histopathological findings. It also highlights the successful resolution through adrenalectomy and supportive care.

**Figure 4:**
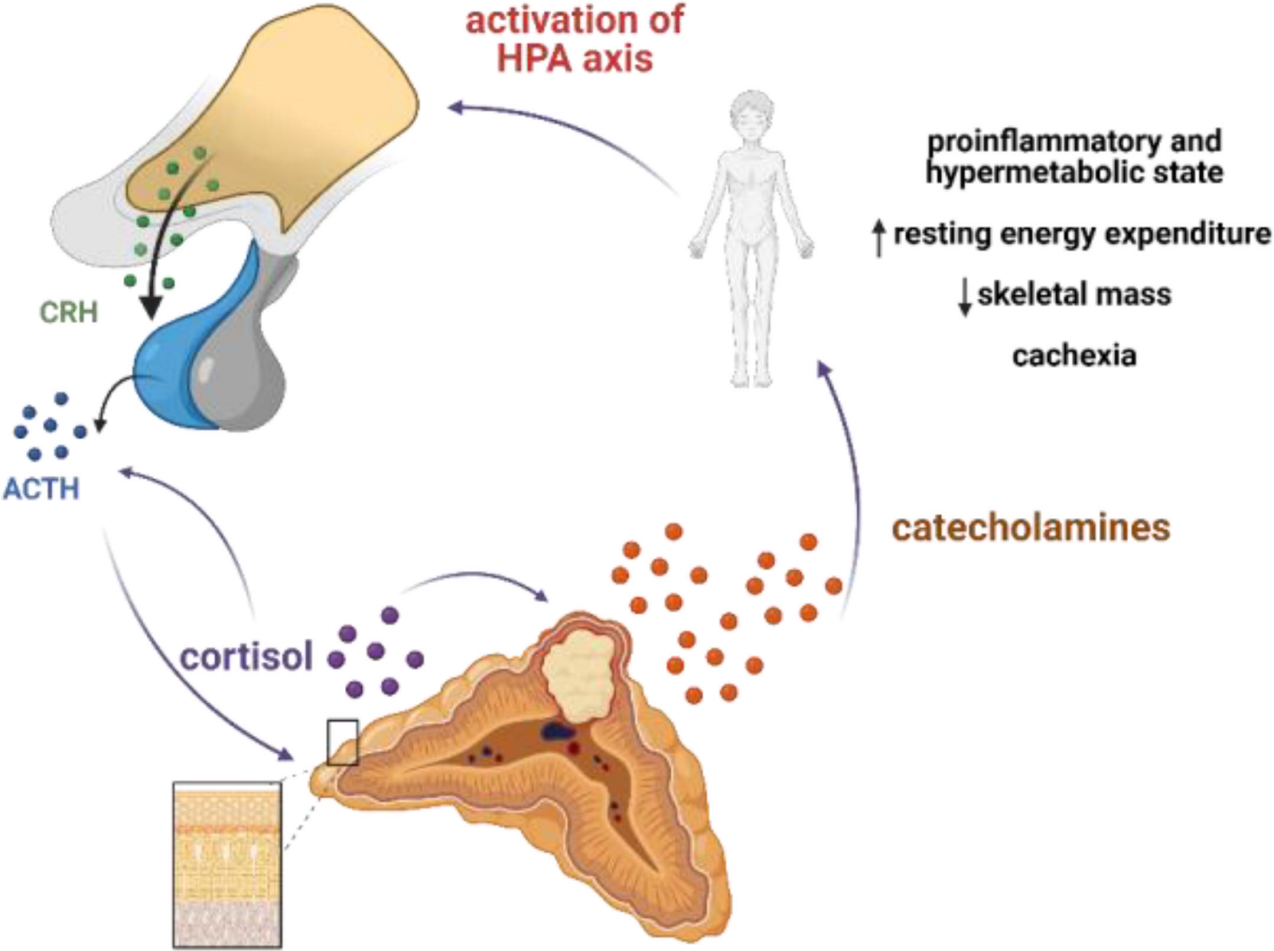
Postulated mechanism of pseudo-Cushing syndrome in our patient. Abundant and chronic secretion of catecholamines leads to hypermetabolic and proinflammatory state that in turn activates HPA axis. Cortisol may potentiate the secretion of catecholamines by increasing the expression of thyrosine hydroxylase (TH), a rate-limiting enzyme in the catecholamine synthesis pathway; and phenylethanolamine N-methyltransferase, a key enzyme in noradrenaline-to-adrenaline conversion. A glucocorticoid-dependent positive-feedback loop may potentiate ACTH release. Created with BioRender.

On admission to the endocrine department, our patient presented with cachexia and no typical symptoms of CS despite significant hypercortisolemia. The reason for such a clinical picture was the chronic overproduction of catecholamines leading to a hypermetabolic state through multiple mechanisms. Hypermetabolism might be mediated directly by adrenoceptors in metabolically active organs and tissues, resulting in stimulation of lipolysis and glycogenolysis, and indirectly, through induction of inflammation, which is reflected by increased secretion of pro-inflammatory cytokines such as IL-1, IL-6, and TNF-α (20). In fact, increased resting energy expenditure (REE) measured by indirect calorimetry, accompanied by increased levels of pro-inflammatory cytokines, has been reported in a cohort of pheochromocytoma patients. Surgical treatment led to a decrease of REE to the expected calculated levels and normalization of some of the inflammation markers (20, 21). Moreover, catecholamines have been shown to activate brown adipose tissue, which was associated with decreased survival (22). Interestingly, a hypermetabolic phenotype of pheochromocytoma is more often reported in the elderly (23), and it can lead to cachexia (24), as in the described patient. In this case, we hypothesize that abundant and chronic secretion of catecholamines from the tumor led to a hypermetabolic and pro-inflammatory state that, in turn, overactivated the HPA axis (**Figure 4**). A rapid decrease in the level of cortisol, ACTH, and metanephrines upon initiation of metyrapone treatment suggests the existence of a glucocorticoid-dependent positive feedback loop that potentiated ACTH release and created a destructive cycle with rapid exacerbation of both hypercortisolemia and hypercatecholaminemia, with extremely elevated plasma ACTH levels. Metyrapone appeared to be clinically effective and resulted in lower levels of cortisol and catecholamines along with significantly lower levels of ACTH.

The biochemical picture of PCS may present as ACTH-dependent CS. It has been proposed that some diagnostic tests may facilitate distinguishing these two diagnoses (5); however, the diagnostic options are limited with no access to CRH. In the case presented here, no dynamic tests were performed due to the severity of the patient’s condition. Some authors suggest that midnight cortisol serum levels above 7.5 μg/dL discriminate CS from PCS with 96% sensitivity and 100% specificity, as in PCS, an unaltered diurnal cortisol rhythm can be observed (25, 26). The disruption of the circadian rhythm of cortisol (and ACTH) is a distinguishing characteristic of CS, however, in our patient, the levels of midnight cortisol were highly elevated despite a lack of ACTH-positive cells in IHC and no other signs of CS. We did not find another case of pseudo-Cushing’s in the literature in which midnight cortisol levels were as high or higher. Disruptions in this rhythm are linked to a wide variety of psychological and physical conditions, such as depression (2), cognitive impairments (27), post-traumatic stress disorder (28), chronic stress (29), burnout (30), chronic fatigue syndrome (31), and anorexia nervosa (32). Upon admission, our patient was in a severe mental state due to discomfort, anxiety, and stress related to hospitalization. It is plausible that psychological factors, combined with cachexia, contributed to the disturbances in nocturnal cortisol secretion. There are no algorithms concerning the treatment of PCS, as cortisol levels are usually normalized following the resolution of the underlying cause. So far, only a few reports address the role of steroidogenesis inhibitors in lowering cortisol and contributing to recovery (6, 33). Our case demonstrates the successful resolution of hypercortisolemia before surgery by the administration of metyrapone. The importance of therapy with steroidogenesis inhibitors lies in their ability to prevent metabolic complications and eliminate glucocorticoid-induced immunosuppression.

## Conclusions

The biochemical picture of PCS may present as ACTH dependent CS. Differentiation of PCS from other causes of hypercortisolemia poses a clinical challenge. Non-neoplastic hypercortisolemia occurs as a response to severe disorders and should always be considered in case of atypical course of hypercortisolemia. Metyrapone might be effective to block the glucocorticoid-dependent positive-feedback loop and minimalize the levels of cortisol, catecholamines, along with ACTH, thus it can reduce the perioperative risk.

## Data Availability

The original contributions presented in the study are included in the article/supplementary material. Further inquiries can be directed to the corresponding author.
