# Corrigendum: Case report: Pheochromocytoma-induced pseudo-Cushing’s syndrome

**DOI:** 10.3389/fendo.2025.1629205

**Published:** 2025-05-29

**Authors:** Małgorzata Bobrowicz, Anna Nagórska, Anna Karpiłowska, Marek Rosłon, Joanna Hubska, Adrianna Gładka, Sadegh Toutounchi, Łukasz Koperski, Urszula Ambroziak

**Affiliations:** ^1^ Department of Internal Medicine and Endocrinology, University Clinical Centre of the Medical University of Warsaw, Warsaw, Poland; ^2^ Doctoral School of Medical University of Warsaw, Warsaw, Poland; ^3^ Department of General, Endocrine, and Vascular Surgery, Medical University of Warsaw, Warsaw, Poland; ^4^ Department of Pathology, Medical University of Warsaw, Warsaw, Poland

**Keywords:** pseudo-Cushing’s syndrome, ectopic ACTH secretion, hypercortisolaemia, pheochromocytoma, cachexia

In the published article, the authors’ names were written incorrectly. The correct spelling is “Małgorzata Bobrowicz, Anna Nagórska, Anna Karpiłowska, Marek Rosłon, Joanna Hubska, Adrianna Gładka, Sadegh Toutounchi, Łukasz Koperski, and Urszula Ambroziak”.

The authors apologize for this error and state that this does not change the scientific conclusions of the article in any way. The original article has been updated.

